# Abrupt peaks in perceived risk of occasional drug use after changing the question order in a repeated self-administered survey

**DOI:** 10.3389/fpubh.2023.971239

**Published:** 2023-04-14

**Authors:** César Pérez-Romero, Gregorio Barrio, Juan Hoyos, María J. Belza, Enrique Regidor, Marta Donat, Julieta Politi, Juan Miguel Guerras, José Pulido

**Affiliations:** ^1^National School of Public Health, Carlos III Health Institute, Madrid, Spain; ^2^The Biomedical Research Center Network for Epidemiology and Public Health (CIBERESP), Madrid, Spain; ^3^Department of Public Health & Maternal and Child Health, Faculty of Medicine, Complutense University of Madrid, Madrid, Spain; ^4^Health Research Institute of San Carlos (IdISSC), Madrid, Spain; ^5^National Centre of Epidemiology, Carlos III Health Institute, Madrid, Spain

**Keywords:** question-order, drug use, risk perception, natural experiment, cognitive heuristics, anchoring

## Abstract

**Background:**

Question-order changes in repeated surveys can distort comparisons. We want to describe the evolution of drug risk perceptions among Spanish adolescents and assessing whether the 2006 peaks in perceived risk of occasional drug use can be explained by question-order changes.

**Methods:**

The subjects were secondary students from a biennial national survey during 2000–2012. A one-off intervention was applied in 2006, replacing the two-adjacent items on perceived risk of occasional and regular use of each drug by non-adjacent items. Annual prevalence of high-risk perception were obtained for occasional and regular use of cannabis, heroin, cocaine and ecstasy. Subsequently, the 2006 percent level change (PC) in such were estimated prevalence using segmented Poisson regression, adjusting for various student and parent covariates.

**Results:**

The 2006 PC in prevalence of high-risk perception of occasional drug use ranged from +63% (heroin) to +83% (ecstasy). These PCs were very high in all considered subgroups. However, the 2006 PC in prevalence of high-risk perception of regular drug use ranged from 1% (heroin) to 12% (cannabis). The evolution of preventive interventions does not suggest alternative causal hypotheses for 2006 peaks other than question-order changes.

**Conclusion:**

Within the cognitive heuristics framework, the 2006 spikes in perceived risk of occasional drug use were most likely due to a release of the anchor exerted by perceived risk of regular drug use over that of occasional use triggered by 2006 question-order changes. In repeated surveys it is inexcusable to pre-test the effect of any change in questionnaire format.

## Introduction

Repeated surveys are often used to make cross-period or cross-site comparisons of health-related phenomena in both patients and population, in order to improve decision-making on health interventions. These surveys are prone to information bias that can severely limit such comparisons, due to various factors, including differences or changes in measurement instruments, particularly questionnaires. When designing survey questionnaires, most researchers know that they must keep the same question wording in different or successive measurements, but they often forget that changes in the format, ordering or clustering of the questions can strongly affect their interpretation and response (1–4). Changing, deleting or adding a previous question item could greatly alter the answers to the next one, even if its wording is entirely kept (5–12), particularly when questionnaires include multiple question items on similar topics with identical response categories –question grids- (13). In some self-administered question grids, a subsequent question may also affect the preceding one, because the respondent may receive both simultaneously (14, 15). These context or question-order effects can appear when recalling witnessed events, behaviors, tasks performance or people (7, 11, 16). However, they are especially conspicuous for subjective phenomena such as perceptions, beliefs, values, preferences, attitudes or future intentions, since they are usually subject to greater uncertainty in response selection (6, 8–10, 15, 17–19). Susceptibility to order effects depends on numerous factors, including the nature and difficulty of the self-reported subject, specific questionnaire format, and respondent factors such as sex, age, education level, or direct experience on the subject (20–23). It is generally believed that the greater is the respondent’s direct experience or personal involvement in the self-reported issue and their cognitive abilities, the less prone they would be to question-order effects. However, previous evidence on the influence of these factors is often inconsistent (23).

Among the subjective phenomena prone to question order effects are people’s risk perceptions on health-related issues, which are relevant in predicting future decisions and actions of people regarding such issues and implementing strategies to control and reduce the associated health and social harm. Thus, question-order effects have been identified when measuring risk perceptions on diseases/causes of death (cancer, COVID-19, homicide), psychiatric patients, environmental hazards (nuclear power, second-hand smoke, air pollution, food preservatives, electromagnetic radiations, traffic accidents) and health-related behaviors (tobacco, alcohol or drug use) (24–32).

Perceived risk of illicit drug use by adolescents is routinely monitored in many countries (33–35), most of these data employed on secondary analysis on risk prevention (36). ESTUDES is a biennial school survey on drugs aimed at secondary school students conducted in all Spain since 1994, which uses a self-administered paper-and-pencil questionnaire, including questions on subjective judgments on risk of occasional and regular use of different illicit drugs (37). In 2006, the ordering and clustering of the questions on perceived risk of occasional and regular drug use was changed. In statistical analyses, sudden increases in risk perception of occasional use of all considered drugs were found in 2006, which were suspected to be artifacts due to the aforementioned reordering of questionnaire items, so in 2008 and later editions it was decided to restore the initial questionnaire format. The study objective is to describe the evolution over time of the perceived risk of occasional and regular use of illicit drugs among Spanish adolescents stratified according to respondent’s sociodemographic factors, and to assess whether the 2006 peaks in perceived risk of occasional drug use can be explained by question-order changes.

## Materials and methods

### Study population

Secondary school students aged 14–18 years who participated in the seven editions of ESTUDES biennial survey during 2000–2012. A national representative sample was selected in each edition through a two-stage cluster sampling procedure (school and classroom) (37). The total sample size was 188,921 students, ranging from 20,450 to 32,234 in the different editions.

### Study design and variables

The study is conceived as a large-scale unplanned single-group experiment, in which a one-off intervention that had not been applied between 2000 and 2004 was applied in 2006 to the entire study population and ceased to be applied in subsequent years, being analyzed the changes in a target and a control outcome over time. The intervention consisted of changing the ordering of questions on drug risk perception (question-order changes), although in fact it also implies a change in their clustering. Specifically, drug risk perception had been assessed until 2006 by an adjacent item-pair model, in which the pair of questions assessing the risk perception of occasional and regular use of a given drug were consecutively presented one after the other without any intermediate question. However, the questionnaire changed in 2006 towards a non-adjacent item-pair model, in which the pair of questions mentioned for a given drug were not consecutively presented, but in two large different blocks or grids, the first referring to occasional use of all drugs and the second to regular use (Table 1).

**Table 1:** Questionnaire models on perceived risk of drug use included in ESTUDES survey (Spain, 2000–2012).

A. Adjacent item-pairs model^1^ (ESTUDES Surveys before and after 2006)					
Grid 1. What do you think about the problems (health or otherwise) that the occasional and regular use of each drug entails.					
	Response options				
	No problem	Few problems	Quite a few	Many	Do not know
Using tranquillizers/sleeping pills occasionally					
Using tranquillizers/sleeping pills regularly					
*Smoking hash/marijuana occasionally*					
*Smoking hash/marijuana regularly*					
Smoking cocaine base or crack occasionally					
Smoking cocaine base or crack regularly					
*Using cocaine occasionally*					
*Using cocaine regularly*					
Using GHB or liquid ecstasy occasionally					
Using GHB or liquid ecstasy regularly					
*Using ecstasy occasionally*					
*Using ecstasy regularly*					
Using speed or amphetamines occasionally					
Using speed or amphetamines regularly					
Using LSD or magic mushrooms occasionally					
Using LSD or magic mushrooms regularly					
*Using heroin occasionally*					
*Using heroin regularly*					
B. Non-adjacent item-pair model^2^ (2006 ESTUDES Survey)					
Grid 1. What do you think about the problems (health or otherwise) that the occasional use of each drug entails.					
	Response options				
	No problem	Few problems	Quite a few	Many	Do not know
Using tranquillizers/sleeping pills occasionally					
*Smoking hash/marijuana occasionally*					
Smoking cocaine base or crack occasionally					
*Using cocaine powder occasionally*						B. Non-adjacent item-pair model^2^ (2006 ESTUDES Survey)
Grid 1. What do you think about the problems (health or otherwise) that the occasional use of each drug entails.					
	Response options				
	No problem	Few problems	Quite a few	Many	Do not know
Using GHB or liquid ecstasy occasionally					
*Using ecstasy occasionally*					
Using speed or amphetamines occasionally					
Using LSD or magic mushrooms occasionally					
*Using heroin occasionally*					
Injecting drugs occasionally					
Grid 2. What do you think about the problems (health or otherwise) that the regular use of each drug entails.					
	Response options				
	No problem	Few problems	Quite a few	Many	Do not know
Using tranquillizers/sleeping pills regularly					
*Smoking hash/marijuana regularly*					
Smoking cocaine base or crack regularly					
*Using cocaine powder regularly*					
Using GHB or liquid ecstasy regularly					
*Using ecstasy regularly*					
Using speed or amphetamines regularly					
Using LSD or magic mushrooms regularly					
*Using heroin regularly*					
Injecting drugs regularly					

^1^It refers to the fact that the questions on risk perception of occasional and regular use of the same drug are presented consecutively one after the other, without any intermediate question. ^2^It refers to the fact that the pair of questions on risk perception of occasional and regular use of the same drug are not presented consecutively one after the other, but in two large different blocks or grids, the first referring to occasional use of all drugs and the second to regular use. Occasionally refers to monthly or less frequently. Regularly refers to weekly or more frequently. *Items in italics* were those analyzed. The questionnaire also included questions on the perceived risk of using other specified substances such as alcohol or tobacco. LSD, GHB, crack, amphetamines and injecting drugs were included for the first time in 2006. Full questionnaires in Spanish are available for consulting in PNSD website (37).

The outcomes were the perceived risk of using occasionally (monthly or less frequently) and regularly (weekly or more frequently) four illicit drugs, specifically cannabis, heroin, cocaine and ecstasy. To assess the risk perception of drug use, the respondents had to indicate the problems (health or otherwise) that entails each of the considered drug use behaviors using an ordinal scale of four responses (no problem, few, quite a few and many) (Table 1). Except for the question-order changes, there were no other changes in questions about outcomes in the analyzed ESTUDES editions, although the questions on cocaine referred simply to “cocaine” until the 2004 edition and “cocaine powder” in the 2006 and later editions (37). For stratification or adjustment the following individual covariates were considered: sex, age, education level, ever had to repeat an annual course, parents’ education level, parents’ employment status, frequency of going out for fun in the evenings as indicator of leisure habits, and use of each considered drug. The education level and course repetition were considered as indicators of academic performance to achieve cognitive skills, and age was used as a proxy of expertise (knowledge on the self-reported subject), since it is assumed that older students have received more information on drug risks (prevention programs, courses, etc.) and have greater capacity to integrate that information. In addition to the ESTUDES variables, data on annual indicators of preventive interventions were obtained from activity reports of the National Plan on Drugs (38), in order to assess if the 2006 changes in outcomes could depend on greater magnitude of such interventions. These indicators include number of schools involved in drug prevention programs, secondary students coverage of such programs, spending index on drug prevention per secondary student and implementation of national preventive campaigns in the media. School drug prevention programs are structured interventions including scheduled sessions (>5) to be developed in the classroom by teachers, or by external prevention experts, often with application manuals, aiming at the development of student’s skills and competencies for life and to avoid drug use. Occasional preventive activities such as talks, distribution of written materials, workshops, awareness days, contests or exhibitions are excluded. The coverage of secondary students by school drug prevention programs is the percentage of total secondary students in Spain (including vocational training) who participated in those programs. The spending index on drug prevention programs per secondary student was calculated by dividing the inflation-adjusted national budget for drug use prevention by the number of secondary students registered in Spain and expressing the result in relation to the year 2000 whose spending per student was assigned the value of 100. The aforementioned budget is the sum of the budgets of the central and regional governments and with it, school and non-school drug prevention interventions are financed. The national preventive campaigns in the media were aimed at informing and sensitizing the Spanish population, especially adolescents aged 12–18 years and their parents or guardians, about the risks of drug use.

### Data analysis

After a descriptive analysis, the prevalences of high-risk perception of occasional and regular use of cannabis, heroin, cocaine and ecstasy for each edition of ESTUDES during 2000–2012 were obtained. We considered “high-risk” perception regarding the use of a given drug when the respondent believed that such behavior could cause many problems. Next, two multivariate approaches were used to assess the magnitude of the 2006 change in high-risk perception of drug use compared to previous and subsequent years. The first approach was to estimate the annual Adjusted Prevalence Ratios (aPRs) of high-risk perception and its 95% confidence intervals (95% CIs) from Poisson regression models with robust variance (39) using the year 2000 as a reference. Adjustment covariates were referred to the students (sex, age, indicators of academic performance, leisure habits, and use or not of assessed drugs) or their parents (parents’ education level, parents’ employment status). The second approach was to use an interrupted time series design, which was analyzed with segmented Poisson regression. This design allowed adjustment for the covariates just mentioned plus the underlying time trend (40, 41). As the intervention seems to cause a temporary change (2006) in the outcome level immediately after the intervention, which disappears in the following years, we have adopted the impact model called “temporary level change” (41). This model can be formalized as Y_t_ = β_0_ + β_1_T_t_ + β_2_X_t_ + β_k_X_k_ + ε, where Y_t_ is the log of annual prevalence, T_t_ a continuous covariate whose value is the number of years elapsed since 2000, X_t_ a binary predictor for the intervention (questionnaire change) with a value of 1 in 2006 and 0 other years, X_k_ a vector for the adjustment covariates other than time, and ε the error term. In this model β_0_ represents the intercept or baseline prevalence level, β_1_ the change in outcome for each unit increase in sequential calendar year (underlying linear time trend for 2000–2012), β_2_ the immediate level change in outcome following the intervention, and β_k_ the coefficients of different adjustment covariates. To facilitate the interpretation, we transformed the β_2_ regression coefficients to relative percent changes (PCs) as 100 (e^β^–1). The 95% CI of PCs were estimated as 100 (e^β ± 1.96SE^–1), where SE is the standard error of β. Analyses were performed using Stata V.14.0 (Stata Corporation, College Station, Texas, USA).

## Results

General characteristics of study participants are shown in Table 2.

**Table 2:** General characteristics of participants in ESTUDES survey by calendar-year (%) (Spain, 2000–2012).

	Year						
	2000	2002	2004	2006	2008	2010	2012
Sample size (n)	20,450	26,576	25,521	26,454	30,183	32,234	27,503
Female (%)	49.1	51.8	50.4	52.4	50.5	51.7	49.5
Age (%)							
14–15 years	40.0	42.6	40.2	44.4	43.9	44.1	34.3
16 years	28.5	25.4	32.4	25.7	26.7	27.9	24.0
17–18 years	31.5	32.1	27.5	29.9	29.5	28.0	41.7
Education level (%)							
Secondary, 1st stage	50.4	53.9	54.1	56.5	51.9	56.3	47.5
Secondary, 2nd stage	38.3	34.2	33.0	36.0	34.3	36.0	36.0
Vocational education	11.4	11.9	12.9	7.5	13.8	7.8	16.6
Ever had to repeat an annual course (%)	35.7	35.3	34.9	33.2	36.8	32.9	32.4
Parents’ education level completed							
At least one parent university education	21.5	24.9	27.0	26.0	25.2	28.8	28.5
At least one parent secondary education	12.9	13.1	13.4	16.2	15.5	19.6	20.1
Both parents < secondary education	40.7	37.9	36.4	36.0	35.3	31.5	32.9
Unknown	24.9	24.1	23.2	21.8	24.1	20.2	18.4
Parents’ employment status							
Both parents employed	47.5	52.0	54.9	53.2	53.3	51.1	46.8
One parent employed	46.5	42.9	40.5	40.1	39.5	38.7	40.0
Both parents unemployed or unknown	6.0	5.2	4.5	6.7	7.2	10.3	13.2
Going out for fun in the evenings ≥ weekly	59.7	54.1	56.7	52.8	56.1	48.6	47.3
Drug use in last 30 days							
Cannabis	20.7	22.5	25.0	20.1	19.8	17.2	16.1
Heroin	0.3	0.2	0.4	0.5	0.5	0.5	0.6
Cocaine	2.5	3.2	3.8	2.0	1.6	1.2	1.1
Ecstasy	2.4	1.9	1.5	1.4	1.0	0.9	1.2

The methods and results of the ESTUDES surveys during the study period can be consulted on the website of the Spanish National Plan on Drugs (37).

### Evolution of prevalence of high-risk perception of drug use during study period

**Figure 1:**
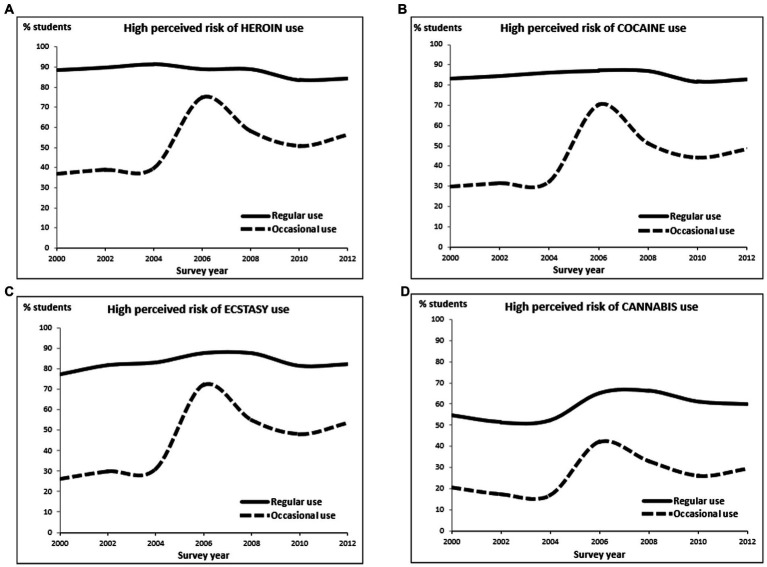
Evolution of the prevalence of high perceived risk of regular or occasional use of heroin **(A)**, cocaine **(B)**, ecstasy **(C)**, and cannabis **(D)** by survey year among secondary students aged 14–18 (%). Spain, 2000–2012. High perceived risk: the student thought that a drug use behavior (for example, using heroin regularly) could cause many problems (in health or other aspects). Regular use: using a drug weekly or more frequently. Occasional use: using a drug monthly or less frequently. The lines are shown smoothed.

The evolution of the prevalence of high-risk perception of drug use was very different for occasional and regular use (Figure 1). For occasional use the prevalence followed a relatively stable trend during pre-intervention period (2000–2004), it increased sharply in the intervention year (2006), doubling their figures, and decreased again in post-intervention period (2008–2012), although maintaining a slightly higher level than in 2000–2004. Thus, the prevalence of high-risk perception was 17–21% in 2000–2004, 42% in 2006 and 26–33% in 2008–2012 for cannabis use, 37–40, 75% and 51–58%, respectively, for heroin use, 30–33, 70% and 44–51% for cocaine use, and 26–31, 72% and 48–55% for ecstasy use. Abrupt spikes in prevalence of high-risk perception of regular drug use during 2006 were not observed. Thus, such prevalences in 2000–2012 ranged 51–65% (cannabis), 84–91% (heroin), 83–87% (cocaine), and 77–88% (ecstasy).

### Impact assessment of 2006 questionnaire change on risk perception of drug use

Like crude prevalences, the aPRs from Poisson regression models showed a very different evolution for occasional and regular drug use (Table 3). Regarding occasional use, the aPR values, using as a reference the prevalence in the year 2000, ranged 0.9–1.2 in pre-intervention years, increased sharply up to 2.0–2.8 in 2006, and decreased again in post-intervention years, staying at a higher level than in 2000–2006 (1.2–2.1). However, the aPRs for regular drug use showed little heterogeneity over time, ranging from 0.9–1.2 during the entire period 2000–2012.

**Table 3:** Adjusted-prevalence ratio of high-risk perception^1^ of drug use by specific drug use behavior and calendar-year [95% confidence intervals] among secondary students aged 14–18 years (Spain, 2000–2012).

	Occasional use				Regular use			
Year	Cannabis	Heroin	Cocaine	Ecstasy	Cannabis	Heroin	Cocaine	Ecstasy
2000	1.0	1.0	1.0	1.0	1.0	1.0	1.0	1.0
2002	0.9 [0.8–0.9]	1.1 [1.0–1.1]	1.0 [1.0–1.1]	1.1 [1.1–1.2]	0.9 [0.9–1.0]	1.0 [1.0–1.0]	1.0 [1.0–1.0]	1.0 [1.0–1.1]
2004	0.9 [0.8–0.9]	1.1 [1.1–1.1]	1.1 [1.1–1.2]	1.2 [1.1–1.2]	1.0 [1.0–1.0]	1.0 [1.0–1.0]	1.0 [1.0–1.1]	1.1 [1.1–1.1]
2006	2.0 [1.9–2.1]	2.1 [2.0–2.1]	2.4 [2.3–2.4]	2.8 [2.7–2.9]	1.2 [1.2–1.2]	1.0 [1.0–1.0]	1.0 [1.0–1.1]	1.1 [1.1–1.1]
2008	1.6 [1.5–1.6]	1.6 [1.5–1.6]	1.7 [1.7–1.8]	2.1 [2.0–2.2]	1.2 [1.2–1.2]	1.0 [1.0–1.0]	1.0 [1.0–1.1]	1.1 [1.1–1.1]
2010	1.2 [1.2–1.3]	1.4 [1.4–1.4]	1.5 [1.4–1.5]	1.8 [1.8–1.9]	1.1 [1.1–1.1]	0.9 [0.9–1.0]	1.0 [1.0–1.0]	1.0 [1.0–1.1]
2012	1.4 [1.4–1.5]	1.5 [1.5–1.6]	1.6 [1.6–1.7]	2.0 [2.0–2.1]	1.1 [1.1–1.1]	1.0 [0.9–1.0]	1.0 [1.0–1.0]	1.1 [1.1–1.1]

^1^They were obtained from Poisson-regression models using 2000 as reference. Results were adjusted by sex, age, indicators of academic performance, leisure habits, use of assessed drugs, parents’ education level and parents’ employment status.

Results from Poisson segmented regression models indicate an upward underlying linear trend in prevalence of high-risk perception of occasional drug use in 2000–2012 with annual PCs of 5, 4, 5, and 7% for cannabis, heroin, cocaine and ecstasy, respectively. Furthermore, there was an immediate relative level change in such prevalence during 2006 of 77, 63, 79, and 83%, respectively (Table 4). However, the 2006 level changes in prevalence of high-risk perception of regular use of cannabis, heroin, cocaine and ecstasy and their corresponding 95% CI were, respectively, 12% (10, 13%), 1% (0, 2%), 3% (2, 4%), and 6% (5, 7%).

**Table 4:** 2006 percent level change in prevalence of high-risk perception of occasional drug use^1^ [95% confidence interval] by subgroups of secondary students aged 14–18 years (Spain, 2000–2012).

	Cannabis	Heroin	Cocaine	Ecstasy
Total	77 [73–81]	63 [61–65]	79 [77–82]	83 [80–86]
Sex				
Men	74 [68–80]	50 [50–56]	72 [68–76]	74 [71–78]
Women	79 [74–85]	72 [69–76]	86 [83–90]	91 [87–95]
Age				
14–15	72 [67–77]	69 [66–73]	80 [76–83]	80 [76–83]
16	79 [70–88]	64 [60–69]	82 [76–87]	88 [83–94]
17–18	85 [75–95]	53 [49–56]	76 [71–81]	82 [77–88]
Education level^2^				
Secondary, 1st stage	71 [67–76]	65 [62–68]	77 [74–80]	78 [75–81]
Secondary, 2nd stage	93 [84–103]	65 [62–69]	86 [81–91]	94 [89–99]
Annual course repetition				
Yes	70 [62–77]	48 [45–52]	69 [64–73]	72 [68–77]
No	80 [75–85]	70 [68–73]	84 [81–88]	88 [85–91]
Use of specific drug assessed in last 30 days^3^				
Yes	189 [159–223]	11 [−30–77]	164 [114–227]	134 [80–205]
No	71 [67–75]	63 [61–65]	78 [76–81]	82 [80–85]

^1^Obtained from segmented Poisson regression by building an impact model which specified a temporary level change during 2006 adjusted by underlying time trend during the entire period 2000–2012 and indicators of academic performance, leisure habits, use of assessed drugs, parents’ education level and parents’ employment status, except stratification variable. ^2^Indicators for vocational training students were not obtained due to the small sample size in this subgroup and its heterogeneous characteristics. ^3^Referred to the use of the specific drug of which risk perception is assessed.

### Stratified analysis of impact assessment of 2006 questionnaire change

Focusing on the results of segmented Poisson regression models, a large 2006 immediate percent level increase in prevalence of high-risk perception of occasional drug use was observed in all subgroups of sex, age and academic performance and in both users and non-users of each assessed drug, except in heroin users where only a very slight non-statistically significant increase was observed. These increases were higher in women than men, although the differences were not statistically significant for cannabis. Likewise, the increases were generally greater in students with higher educational level or higher academic performance (as students in the second stage of secondary education or students who had not repeated any complete annual course), except for heroin. Regarding age, no consistent results were observed for the different drugs. Thus, the magnitude of the immediate level increase increased with age for cannabis, decreased with age for heroin, and varied little for cocaine and ecstasy (Table 4).

### Evolution of preventive drug use interventions in Spain during study period

The evolution of four indicators of drug prevention interventions aimed at secondary students is shown in Table 5.

**Table 5:** Evolution of indicators on drug prevention interventions implemented by the public administration in Spain, 2000–2012.

Year	Schools involved in drug prevention programs^1^ (N° schools in 2000 = 100)	Coverage of secondary students by school drug prevention programs^2^ (%)	Spending index on drug prevention per secondary student^3^ (2000 spending = 100)	National preventive campaigns in the media^4^
2000	100	20.7	100	Yes
2001	129	18.9	92	Yes
2002	129	26.1	149	Yes
2003	192	33.3	166	Yes
2004	184	38.6	200	Yes
2005	201^5^	42.4	201	Yes
2006	230	55.6	226	Yes
2007	149	42.9	223	Yes
2008	221	58.7	225	No
2009	209	50.7	208	No
2010	251	54.1	204	No
2011	166	45.7	170	No
2012	178	40.5	115	No

^1^Referred to schools with structured drug prevention programs aimed at secondary students (including vocational training). Such programs are those with scheduled sessions (>5) to be developed in the classroom by teachers, or by external prevention experts, often with application manuals, and aimed at the development of skills and competencies for life or to avoid drug use. Occasional preventive activities such as talks, distribution of written materials, workshops, awareness days, contests or exhibitions are not considered. ^2^This refers to the percentage of total students enrolled in secondary education in Spain (including vocational training) who participated in structured drug prevention programs. ^3^It was calculated by dividing the inflation-adjusted national budget for drug use prevention by the number of secondary students registered in Spain and expressing the result in relation to the year 2000 whose spending per student was assigned the value of 100. The aforementioned budget is the sum of the budgets of the central and regional governments and with it, school and non-school drug prevention interventions are financed. ^4^The national preventive campaigns in the media were aimed at informing and sensitizing the Spanish population, especially adolescents aged 12–18 and their parents or guardians, about the risks of drug use. ^5^This data was estimated. Data source: Data were extracted from activity reports of National Plan on Drugs.

Three of those indicators (number of schools involved in drug prevention programs, coverage of secondary students by these programs, and spending index on drug prevention per secondary student) followed a similar evolution, with a significant increase between 2000 and 2006, a relative stabilization between that year and 2010 and a subsequent rapid decrease. For example, the coverage of the school drug prevention programs went from 20.7% in 2000 to 55.6% in 2006, 58.7% in 2008, 54.1% in 2010, and 40.5% in 2012. There were annual campaigns in the general media aimed at drug prevention in 2000–2007, but they disappeared in 2008–2012. The maximum relative interannual increases in such indicators were found in 2002 for the coverage of school drug prevention programs (38.0%), in 2003 for the number of schools involved in drug prevention programs (48.8%), and in 2002 for the spending index on drug prevention per secondary student (62.9%). The 2006 relative annual increase of the different interventions ranged from 12.1% for the spending index on drug prevention per secondary student to 31.3% for the coverage of the school drug prevention programs. The absolute maximums of these indicators were in 2008, 2010, and 2006, respectively. Moreover, parents’ and teachers’ involvement in drug prevention increased in 2006–2010 (data not shown in Table 5).

## Discussion

In 2006, after a question-order change in ESTUDES survey, there was a sudden and large increase in risk perception of occasional use of cannabis, heroin, cocaine and ecstasy among Spanish adolescents, which largely disappeared in 2008 after restoring the initial questionnaire format. Thus, the relative percent changes in prevalence of high-risk perception immediately after the 2006 intervention ranged from +63% (heroin) to +83% (ecstasy). These increases occurred in all sociodemographic subgroups analyzed. However, the 2006 increases in perceived risk of regular use of these drugs were very small, with relative percentage changes in prevalence of high-risk perception ranging from +1% (heroin) to +12% (cannabis). After assessing the evolution of preventive interventions, no sudden increases in magnitude of these interventions were identified in 2006, which does not suggest alternative causal hypotheses for 2006 peaks in perceived risk of occasional drug use other than 2006 question-order changes.

### Question-order changes as the main cause of the 2006 peaks in drug risk perception

It is highly likely that the 2006 peaks in the risk perception of occasional drug use was mainly due to question-order changes for several reasons. First, it was a peak of great magnitude, located only in 2006 and that disappeared after restoring the initial questionnaire format. Second, the Poisson regression model that identified the peak was adjusted for several individual time-varying covariates to avoid confounding. Third, the segmented Poisson regression model to measure the 2006 immediate relative change in prevalence level of high-risk perception was further adjusted for the underlying linear time trend during 2000–2012, which implies some control for unmeasured individual time-varying confounders that change slowly over time (42). Fourth, the peak was identified in all sociodemographic subgroups analyzed, including those of sex, age, education level and academic performance, both in drug and non-drug users (except heroin users). Fifth, the peak was almost non-existent for the perceived risk of regular drug use, considered the control outcome or negative control. Sixth, in 2006 there were no abrupt increases in magnitude of preventive drug use interventions. Thus, although 2006 had the largest budget for drug prevention during the analyzed period, no prevention indicator had in 2006 its highest annual percent increase. Furthermore, the 2006 annual percent increases in prevention indicators ranged 12–31% compared to 63–83% in the outcome. Although local and regional preventive drug interventions are difficult to measure, the occurrence of sudden large-scale synchronized increases in such interventions in 2006 are highly unlikely, mainly due to their partial dependence on the national budget. Finally, among adolescents an inverse relationship between drug risk perception and drug use is usually observed (43–47). However, in 2006–2010 in Spain the apparent huge decreases in perceived risk of cannabis, cocaine and ecstasy were accompanied by decreases in annual and monthly prevalences of use of these drugs (37), suggesting that decreases in perceived risk were not real. Taken together, the evidence suggests a causal relationship between the 2006 questionnaire change (intervention) and the sudden increases in perceived risk of occasional drug use (outcome).

### Interpreting the 2006 spike in drug risk perception as an anchor release effect

Although there are several explanations on the mechanism of the question-order effects, perhaps the most accepted rely on cognitive heuristics (13, 23). The mental processes of answering questionnaires usually has various steps (question understanding, retrieving relevant factual data from memory, integrating it into a judgment and answer selection) (1, 48). However, when there is considerable uncertainty, as often happens with subjective phenomena such as health-related risk perceptions, especially under conditions of fatigue or disinterest, respondents often resort to heuristics or mental shortcuts to answer by reducing the cognitive burden. One of the best known is the anchoring-and-adjustment heuristic, which involves initially focusing on an anchor or reference point, which is usually the phenomenon better known, easily recalled from memory, or more salient, and then adjusting the selected answer from the anchor (49–54). This heuristic aligns well with the evidence that human judgements are generally comparative (55), and its effects are extremely ubiquitous across topics, subgroups, and settings. Specifically, in a question grid including several items on a similar topic with a similar response scale, sometimes the response to an adjacent item, which is easier to elaborate, is used by respondents as a self-generated anchor to adjust the response to another item either by reducing the difference between both responses (assimilation) or expanding it (contrast) (51, 56).

The adolescents’ judgments on risk of a given drug are subject to great difficulty and uncertainty because most of them could not base such judgments on personal experience since they are non-users or very sporadic users. Consequently, they would resort to anchoring heuristics, integrating the most easily available external information, which usually refers to regular drug use, since it is a very notorious and quite frequently associated behavior with social or health problems, and subsequently adjusting the response on the risk of occasional use from that reference. As indicated, the 2006 questionnaire change consisted of asking on perceived risk of occasional and regular use of a given drug using two non-adjacent items located in different blocks (non-adjacent item-pair model) instead of two adjacent or consecutive items as in 2000–2004 and 2008–2012 (adjacent item-pairs model). The findings show a 2006 peak in perceived risk for occasional drug use but not for regular use. This suggests that in the adjacent item-pairs model, operative in years other than 2006, the risk perception of regular drug use would act as an anchor item (57), so that adolescents would adjust the response to the immediately preceding item on the risk of occasional use, trying to considerably reduce the risk attributed to regular use (contrast effect) (58). However, in the non-adjacent item-pair model, operative in 2006, the anchor of regular use disappears since this item is far away in another question grid, so the perceived risk of occasional drug use shoots up. In short, the 2006 peak in perceived risk of occasional drug use was most likely due to a release from the anchor exerted by the perceived risk of regular drug use. Such release would have been triggered by the 2006 change in question-order.

As indicated, some respondent factors such as sex, age, education level, or direct experience on the subject (20–23) can modify susceptibility to question-order effects. The higher 2006 peaks in risk perception of occasional drug use among women than men found in our research are consistent with previous findings (20) and with an increased women’s susceptibility to anchoring heuristics (59). Regarding age, our results referring to question-order effects in perceived risk of occasional drug use are mixed, since they show a greater effect among older and younger adolescents, respectively, for cannabis and heroin, and almost no effect for cocaine and ecstasy. However, there is some previous finding indicating that younger adolescents are more strongly influenced by questionnaire format and context than older adolescents (22). Finally, the higher peaks in adolescents with higher than lower educational level or academic performance are difficult to interpret. Thus, although it is usually thought that the greater the cognitive ability of the respondent and their direct involvement or experience in a self-reported issue, the less propensity they will have for question order effects, the truth is that the previous evidence in this regard is usually inconsistent (23). Similarly, it would be expected that the higher the educational level and academic performance of an adolescent, the greater their knowledge on drug effects and greater cognitive ability to develop independent responses without resorting to anchors. However, the evidence on susceptibility to anchoring effect according to education or intelligence level is also inconsistent (4, 21, 60–63). The key to the inconsistencies may be that, although the anchoring heuristic can generate bias, it is also a cognitive resource which in situations of great uncertainty and time constraints helps build responses and judgments (64). Lacking conclusive evidence or social consensus on the risk of occasional use of drugs such as cannabis or ecstasy, the adolescents’ judgments on this involve great uncertainty and cognitive burden. Thus, it may be even more rational and valid to adjust the response from reliable anchors than to elaborate it ex novo relying on one’s own beliefs or those of peers.

## Limitations

Given that this study is based on a natural experiment, its main limitation is the potential influence of uncontrolled factors changing between 2006 survey and the others. Notwithstanding, even if it cannot be completely ruled out, the role of potential unknown events (social, political, communicative, etc.) occurring in 2006 is quite unlikely. A detailed search has been made and no one relevant has been found. Other changes in survey methodology (i.e., sampling, fieldwork or database preparation) which could explain the results have not been identified either. In this sense, it is a guarantee that there have not been 2006 abrupt changes in other sociodemographic indicators, drug use, opinion and perceptions from the survey.

On the other hand, dissimilar results observed by drug and sociodemographic subgroup could be argued as a limitation. In fact, these findings do not match with previous knowledge concerning individual susceptibility to cognitive heuristics. However, this background is moderately inconsistent, and this study was not focused on analyzing individual differences. In general terms, the anchoring effect release in 2006 was observed among all drugs and subgroups, and just the magnitude of this fact changed from some groups to others.

## Conclusions

The findings suggest that when assessing the magnitude of a subjective health-related phenomenon subject to considerable uncertainty (i.e., the perceived risk of occasional and regular drug use) using two or more consecutive or adjacent items, responses may strongly depend on question ordering. Consequently, in repeated or panel health surveys, some seemingly “cosmetic” changes in ordering or clustering of questions, particularly the insertion of intermediate items, may cause unexpected anomalies or artifacts that severely limit comparisons. The standardization over time, space, groups and individuals of data collection methods or instruments, including questionnaires, is essential to achieve valid estimates of any health measure, particularly subjective ones, and to be able to make valid comparisons based on them. Questionnaire changes should be minimized and if unavoidable they should be carefully pretested through a cognitive interview and piloted before launching (65, 66). Likewise, researchers and decision makers in the health field must be aware that the comparison of findings from two questionnaires with items with the same wording but different ordering or clustering may be affected by question-order biases that invalidate such a comparison. Another important corollary is that the transfer of evidence from self-reported surveys to policies must be very careful and prudent, since the results are highly dependent on the characteristics of the data collection instrument. For this reason, it is also convenient to triangulate the results of these surveys with other sources or methods, such as qualitative techniques.

## Data availability statement

The raw data supporting the conclusions of this article will be made available by the authors, without undue reservation.

## Ethics statement

Ethical review and approval was not required for the study on human participants in accordance with the local legislation and institutional requirements. Written informed consent to participate in this study was provided by the participants’ legal guardian/next of kin.

## Author contributions

CP-R analyzed the data and wrote the manuscript. JoP and GB originated and designed the study and coordinated the writing of the article. JH, JuP, MD, and MB contributed to the analysis of the data and to the drafting of the manuscript. JG collaborated in the bibliographic search and in the debugging of the database. ER contributed to the interpretation of the results and to the drafting of the manuscript. All authors have contributed to the work, agree to the order in which they are listed, have read and reviwed the final manuscript and approved the final version and its submission.

## Funding

This research was carried out in the framework of a project funded by the Government Delegation for the National Drugs Plan (DGPNSD) (Grant 2019I017).

## Conflict of interest

The authors declare that the research was conducted in the absence of any commercial or financial relationships that could be construed as a potential conflict of interest.

## Publisher’s note

All claims expressed in this article are solely those of the authors and do not necessarily represent those of their affiliated organizations, or those of the publisher, the editors and the reviewers. Any product that may be evaluated in this article, or claim that may be made by its manufacturer, is not guaranteed or endorsed by the publisher.

## Abbreviations

aPR: adjusted prevalence ratio
ESTUDES: Survey on Drug Use in Secondary Education in Spain [Encuesta sobre Uso de drogas en Enseñanzas Secundarias en España]
95% CI: 95% confidence interval
PC: relative percent change
